# Quantitative Trait Locus Mapping Methods for Diversity Outbred Mice

**DOI:** 10.1534/g3.114.013748

**Published:** 2014-09-01

**Authors:** Daniel M. Gatti, Karen L. Svenson, Andrey Shabalin, Long-Yang Wu, William Valdar, Petr Simecek, Neal Goodwin, Riyan Cheng, Daniel Pomp, Abraham Palmer, Elissa J. Chesler, Karl W. Broman, Gary A. Churchill

**Affiliations:** * The Jackson Laboratory, Bar Harbor, Maine 04609; † Medical College of Virginia of Virginia Commonwealth University, Richmond, Virginia 23298; ‡ Department of Genetics, University of North Carolina, Chapel Hill, North Carolina 27599; § Lineberger Comprehensive Cancer Center, University of North Carolina, Chapel Hill, North Carolina 27599; †† Department of Human Genetics, University of Chicago, Chicago, Illinois 60637; ‡‡ Department of Biostatistics and Medical Informatics, University of Wisconsin, Madison, Wisconsin 53706; ** Division of Plant Sciences, Research School of Biology, The Australian National University, Canberra, Australian Capital Territory 0200, Australia

**Keywords:** diversity outbred, haplotype reconstruction, quantitative trait locus mapping, Multiparent Advanced Generation Inter-Cross (MAGIC), multiparental populations, MPP

## Abstract

Genetic mapping studies in the mouse and other model organisms are used to search for genes underlying complex phenotypes. Traditional genetic mapping studies that employ single-generation crosses have poor mapping resolution and limit discovery to loci that are polymorphic between the two parental strains. Multiparent outbreeding populations address these shortcomings by increasing the density of recombination events and introducing allelic variants from multiple founder strains. However, multiparent crosses present new analytical challenges and require specialized software to take full advantage of these benefits. Each animal in an outbreeding population is genetically unique and must be genotyped using a high-density marker set; regression models for mapping must accommodate multiple founder alleles, and complex breeding designs give rise to polygenic covariance among related animals that must be accounted for in mapping analysis. The Diversity Outbred (DO) mice combine the genetic diversity of eight founder strains in a multigenerational breeding design that has been maintained for >16 generations. The large population size and randomized mating ensure the long-term genetic stability of this population. We present a complete analytical pipeline for genetic mapping in DO mice, including algorithms for probabilistic reconstruction of founder haplotypes from genotyping array intensity data, and mapping methods that accommodate multiple founder haplotypes and account for relatedness among animals. Power analysis suggests that studies with as few as 200 DO mice can detect loci with large effects, but loci that account for <5% of trait variance may require a sample size of up to 1000 animals. The methods described here are implemented in the freely available R package DOQTL.

QUANTITATIVE trait locus (QTL) mapping is an approach widely used for detecting and localizing genetic variants responsible for phenotypic variation. Traditionally, QTL intervals are defined by the statistical association of phenotypes with genetic markers in backcross or intercross progeny derived by crossing two inbred strains (Lander and Botstein 1989). This experimental design relies on a single generation of meiotic recombination to break up and randomize the parental genomes; as a result QTL intervals are often too large to identify causal variants (Flint *et al.* 2005). Furthermore, the potential for discovery of genetic associations is limited to loci that are polymorphic between the two parental inbred strains. Experimental mapping populations with increased mapping precision and greater genetic diversity are desirable to improve the performance of QTL mapping studies.

Heterogeneous stocks (HS) derived from multiple inbred founder strains and maintained as outbreeding populations for many generations can increase mapping precision and introduce additional genetic diversity (Li and Lumeng 1984; Hitzemann *et al.* 1994; Iancu *et al.* 2010). The Diversity Outbred (DO) mice are a heterogeneous stock derived from eight inbred founder strains and maintained by randomized breeding among 175 mating pairs (Svenson *et al.* 2012). The founder strains A/J, C57BL6/J, 129S1/SvImJ, NOD/ShiLtJ, NZO/HlLtJ, CAST/EiJ, PWK/PhJ, and WSB/EiJ (denoted below as A–H, respectively) are the same strains that were used to establish the Collaborative Cross (CC) recombinant inbred lines (Churchill *et al.* 2004; Collaborative Cross Consortium 2012). The DO population captures at least 37.8 million single nucleotide polymorphisms (SNPs) plus 6.9 million insertions, deletions, and structural variants that are uniformly distributed across the genome (Keane *et al.* 2011). Each DO mouse is heterozygous by ancestry over an average of 7/8th of its genome, and genetic variants have an expected minimum minor allele frequency (MAF) of 1/8. A related heterogeneous stock (CC-HS) was derived from the same founder strains using a circular breeding design (Iancu *et al.* 2010). The CC, DO, and CC-HS populations segregate the same genetic variants in different population structures; they provide a complementary set of resources for high-resolution QTL mapping and validation.

QTL mapping analysis in HS populations presents a number of analytical challenges. The genome of each animal is a unique mosaic of founder haplotypes. Accumulation of meiotic recombination events over many generations of breeding results in small haplotype blocks that provide high mapping resolution but also require high-density genotyping (>50K SNPs) to reconstruct the haplotypes. We demonstrate the necessity of high-density genotyping to obtain accurate haplotype reconstructions and show that accuracy is improved by directly utilizing probe intensity data from genotyping arrays rather than genotype calls. We produce probabilistic estimates of founder haplotypes and these provide a basis for genetic mapping and genome-wide SNP imputation. QTL mapping regression models can be formulated in a variety of ways using founder haplotypes or SNP allele calls and we implement three different regression models and discuss their relative merits. Unlike simple cross designs, HS mice are not all related to the same degree and covariance among related individuals necessitates the use of a mixed-model linear regression with a kinship term (Kang *et al.* 2008; Cheng *et al.* 2010; Cheng and Palmer 2013). Methods for determining genome-wide significance levels and support intervals for QTL location also require special attention in the context of HS populations. We evaluate the performance of a simple permutation test for HS populations and demonstrate that it has the correct size despite the presence of population structure. Simulations provide estimates of power and sample size required for successful QTL mapping. We illustrate novel features of the DOQTL software using an example of mapping hematological traits in DO mice.

We have implemented specialized QTL mapping software (DOQTL) for use with DO mice and other HS mapping populations. DOQTL implements a complete analytical pipeline for haplotype reconstruction and QTL mapping in the DO. It includes alternative modes of analysis for some steps in the pipeline. The modular design of the software will enable development and evaluation of new methods and extension to other HS populations with relatively few modifications.

## Materials and Methods

### Mice and genotyping

We isolated DNA using the Promega (Madison, WI) Maxwell 16 and Tissue DNA purification kit according to the manufacturer’s instructions. GeneSeek (Lincoln, NE) carried out genotyping assays on a total of 4542 DO mice. Genotype calls—A, B, H, or N—were generated using Illumina’s BeadStudio algorithm. Here A represents homozygosity for the reference allele, B is homozygous for the alternative allele, H is a heterozygous genotype, and N is “no call.”

We genotyped 3029 of the 4542 DO mice from outbreeding generations G4 through G9 using the Mouse Universal Genotyping Array (MUGA) (Welsh *et al.* 2012). The MUGA assays 7854 SNPs spanning the 19 autosomes and X chromosome of the mouse with a mean spacing of 325 kb. We removed 190 probe pairs from the MUGA data that did not perform well. Of these DNA samples, 34 were run in duplicate in different batches of MUGA genotyping.

We genotyped 1513 of the 4542 DO mice from outbreeding generations G8 through G11 using the higher-density MegaMUGA platform (GeneSeek, 2013). The MegaMUGA assays 77,818 SNPs across the mouse genome with a mean spacing of 33 kb. We used a set of 57,977 SNP assays that distinguish among the genotypes of the eight founder strains of the DO and their heterozygous F1 offspring.

We obtained MUGA and MegaMUGA data for the eight founder strains and most of their F1 hybrid progeny. We adjusted the founder and DO sample intensities for batch differences by quantile normalization across samples at each marker and calculated the intensity quantiles at each marker for the DO samples and the founders/F1’s separately. We then normalized the intensities of the founder and F1 samples to match the quantiles of the DO samples.

**Figure 1:**
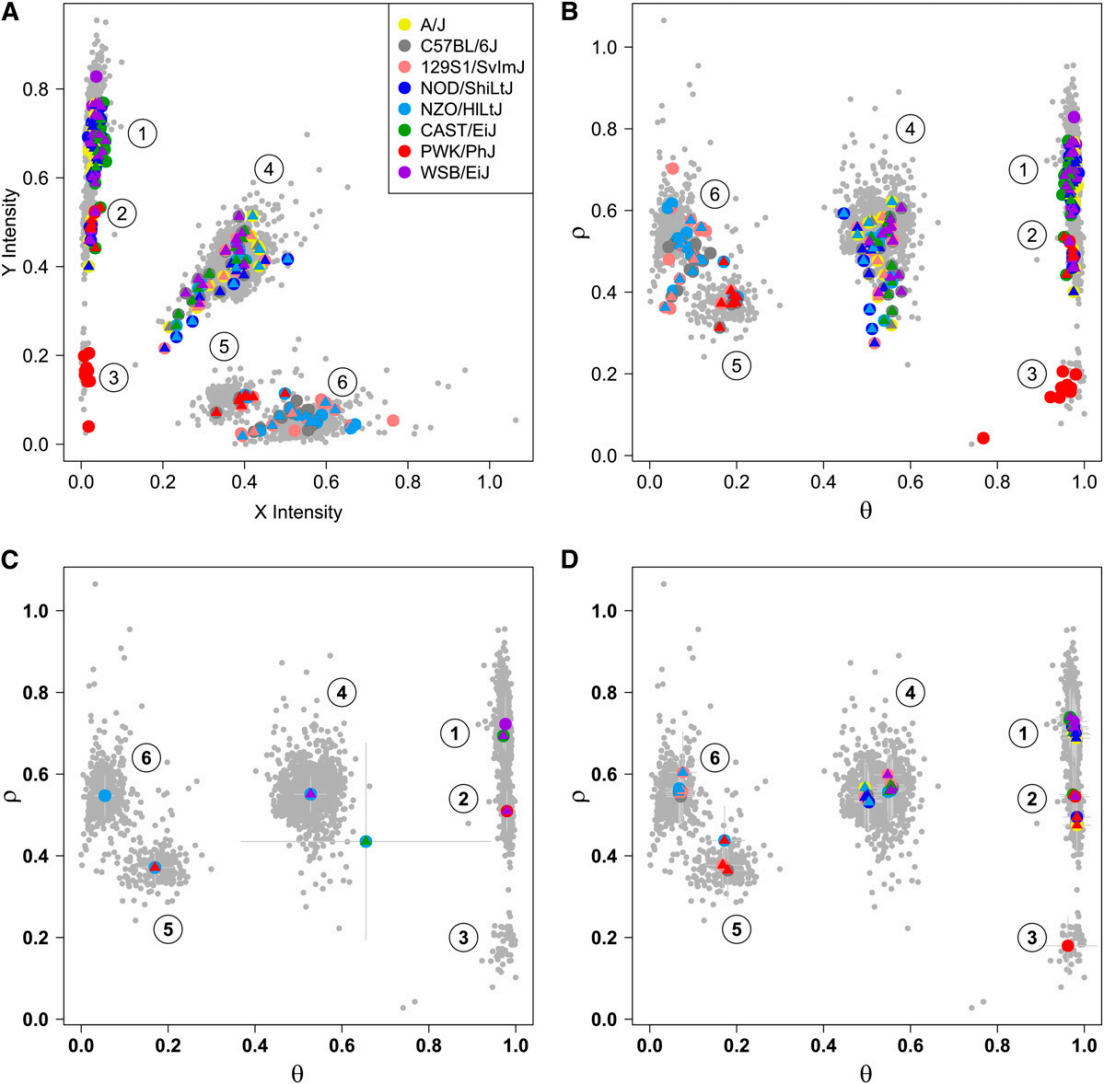
*X and Y* probe intensities on the MUGA contain information about founder haplotypes that is not captured by the four genotype allele calls. Each point represents the *X* and *Y* intensity values for one sample at marker UNC190139327, chromosome 19, 5.083353 Mb (rs46230775). Locations of the founder strains, including multiple independent samples of founder and F1 hybrids animals, are shown as colored circles. Founder strains are represented as colored circles and F1 hybrids are represented by a circle in one founder color and an internal triangle in the other founder color. For example, the eight red points in cluster 3 represent replicates of the PWK/PhJ founder. (A) Cluster 1 represents homozygotes and heterozygotes between A/J, CAST/EiJ, NOD/ShiLtJ, and WSB/EiJ. Cluster 2 represents heterozygotes between the founders in cluster 1 and PWK/PhJ. Cluster 3 represents PWK/PhJ homozygotes. Cluster 4 contains heterozygotes between the founders in clusters 1 and 6. Cluster 5 represents homozygotes and heterozygotes between PWK/PhJ and the strains in cluster 6. Cluster 6 consists of homozygotes and heterozygotes between 129S1/SvImJ, C57BL/6J, and NZO/HlLtJ. (B) The *X* and *Y* intensities from A have been transformed to *ρ* and *θ* coordinates and the cluster axes are aligned vertically. (C) Initial 36 diplotype state cluster locations that are estimated using mclust from the sample and founder data. Many diplotype states have the same cluster center. (D) Final diplotype state locations after the genotyping algorithm has completed.

To obtain reliable and robust reference points for interpreting the intensity data, we assayed multiple DNA samples from animals of each sex, each founder strain, and most of the F1 hybrids, on both MUGA and MegaMUGA platforms. At each marker, we applied a polar coordinate transformation to the *X* and *Y* intensity values on combined data from founder, F1 and DO DNA samples (Figure 1, A and B). We estimated the mean and variance of the angle *θ* and radius *ρ* for each cluster using mclust software (Fraley 2012). Mclust fits a bivariate Gaussian mixture model at each marker and maximizes the Bayesian information criterion to determine the number of clusters. Probe intensities are expected to fall into three distinct clusters corresponding to SNP genotypes A, H, and B. However, 16% of MUGA probes and 6% of MegaMUGA probes consistently yielded more than three distinct clusters due to variant(s) that are present near or within the constant portion of a probe’s target sequence. Intensity data from these nonconforming probes can provide more information than genotype calls leading to improved accuracy in haplotype reconstructions (Fu 2012).

### Haplotype reconstruction

Each chromosome pair of a DO animal is composed of a unique mosaic of founder haplotypes. We refer to the pair of founder haplotypes at a given locus as the founder *diplotype*. There are 36 possible diplotypes—8 homozygous and 28 heterozygous diplotypes. We developed a hidden Markov model (HMM) to reconstruct the diplotypes from the genotype array data by generating a probabilistic estimate of the diplotype state (*s*) at each marker locus (*j*) in each DO animal (*i*). We denote these *diplotype probabilities* as *p_ij_(s)*.

The HMM is composed of a *transition model* and an *emission model*. The transition model is a Markov chain with 36 states that defines the probability of transitioning from one diplotype state to another between adjacent marker loci by meiotic recombination. We calculated transition probabilities between markers based on genetic map positions of markers (Cox *et al.* 2009) and the outbreeding generation (Broman 2012). The emission model defines the conditional probability distribution of observed data given the underlying diplotype state. We implemented two different emission models, a discrete model for genotype call data, and a continuous model for intensity data. Emission model parameters are estimated using an expectation-maximization (EM) algorithm. We describe each of the emission models here.

#### Genotype model:

The emission model for genotype calls is a multinomial distribution over the four outcomes A, H, B, and N. The expected diallelic genotypes corresponding to the diplotype states at a given marker were obtained from the genomic sequences of the founder strains (Keane *et al.* 2011). We initially set the expected genotype to occur with high probability (0.97) and distributed the remaining probability evenly over the other outcomes (0.01 each) to allow for genotype calling errors and no calls. We treat no call N as a separate, informative outcome because it contains information about the underlying diplotype state. A no call can occur at different rates for different true diplotype states and therefore contains information about the diplotype state. For example, when the diplotype is FF (CAST homozygous), the genotype call may be N 90% of the time, whereas for a BB diplotype (C57BL/6J homozygous), N calls may occur only 1% of the time.

#### Intensity model:

The emission model for intensity data are a bivariate Gaussian distribution. For each diplotype state at a given marker, the distribution of intensities in polar coordinates is defined as the product of two univariate normal distributions. We also investigated models with a covariance term and models of *x–y* intensity but these are not considered further here. There are four parameters for each diplotype at each marker, two means and two variances. It is important to obtain good initial estimates for these parameters. At a given marker, we assigned each of the 36 diplotype states to a cluster (described above) using the founder and F1 samples as reference points. Initial parameter estimates for each diplotype state were obtained from these clusters (Figure 1C). However, we allowed each diplotype state to follow its own distribution to correct errors in the initial cluster assignments and to extract additional information from the intensity data (Figure 1D).

#### The EM algorithm:

EM algorithms for parameter estimation in discrete or continuous outcome HMMs have been described in detail elsewhere (Baum 1970; Churchill 1989; Rabiner 1989). In our implementation, transition model parameters were held fixed over iterations; only emission model parameters were estimated from the data. In principle, the transmission parameters could also be estimated but we found that the resulting diplotype reconstructions tended to transition too frequently between diplotype states.

The E-step uses current estimates of model parameters to execute a forward–backward HMM algorithm and generates diplotype probabilities *p_ij_*(*s*). The E-step is computationally intensive but it can be applied to one individual and one chromosome at a time. The M-step uses the diplotype probabilities to compute new estimates of the (emission) model parameters. The M-step can be computed one marker at a time; it combines information across individuals. These steps are iterated until a convergence criterion is satisfied. We stopped iterations when the log-likelihood of the model changed by <1/1000 of the initial log-likelihood in successive iterations. Best results are obtained with large collections of individuals, at least several hundred.

A *marginal reconstruction* of DO haplotypes can be obtained by assigning each locus to the diplotype state that has maximum probability. The diplotypes are phased to obtain a pair of haplotypes by minimizing the number of recombination events. Phasing is not critical for QTL mapping but we use phased chromosomes for graphical representation and to count recombination events. Our phasing algorithm could result in undercounting of recombination events but it is simple and appears to work well. We place transitions at the midpoint between flanking marker loci. SNP genotypes across the entire genome can be imputed from the marginal reconstruction by reference to the founder genome sequences to obtain a diploid genome sequence for each DO animal. Probabilistic imputation of SNP genotypes can also be obtained as discussed below.

### Regression modeling

We computed genome scans by regression on diplotype probabilities (Haley and Knott 1992). We fit a linear mixed model with sex and batch as covariates. A random-effect term captures the polygenic covariance due to kinship among the animals. The likelihood ratio comparing this model to a null model without the diplotype term is converted to a LOD score or to −log10(p-value). We consider three different ways to include the diplotype states in the regression model, which we refer to as the *full model*, the *additive haplotype model*, and the *additive SNP model*. The full and additive haplotype models can be computed at the marker loci or, if desired, at points between marker loci using interpolation to approximate the diplotype probabilities. The additive SNP model is computed at imputed SNP loci.

#### Full model:

The full model is an unconstrained regression on the 36 diplotype states. We express the full model at marker locus *j* as

$$y_{i}=\sum_{k}x_{ik}a_{k}+\sum_{s=1}^{36}p_{ij}(s)\beta_{s}+\gamma_{i}+\varepsilon_{i},$$ (1)

where *y_i_* is the phenotype for animal *i*, *x_ik_* is an indicator for covariate *k* and animal *i*, *a_k_* is the effect of covariate *k*, *p_ij_(s)* is the diplotype probability at a marker locus *j* for animal *i* generated by the HMM, *γ_i_* is an adjustment for kinship, and *ε_i_* is the residual error. The model includes 36 regression coefficients, *β_s_*, one for each diplotype state *s*. The full model is general and allows for arbitrary patterns of additive and dominance effects among the founder alleles. However, the likelihood-ratio test with 35 degrees of freedom is expected to perform poorly unless sample sizes are very large.

#### Additive haplotype model:

The additive model assumes that the effects of founder alleles combine additively; there are no dominance effects in this model. To implement the additive model, we compute the *allelic dosage* of founder *h* at each marker at marker locus *j* as

$$d_{ij}(h)=\sum_{s=1}^{36}p_{ij}(s)N_{h}(s),$$ (2)

where *N_h_(s)* is the number of alleles coming from founder *h* in diplotype *s*. For example, *N_c_*(CC) = 2, *N_c_*(BC) = 1, and *N_c_*(BB) = 0.

We express the regression equation as

$$y_{i}=\sum_{k}x_{ik}a_{k}+\sum_{h=1}^{8}d_{ij}(h)\beta_{h}+\gamma_{i}+\varepsilon_{i},$$ (3)

where *y_i_*, *x_ik_*, *a_k_*, *γ_i_*, and *ε_i_* are the same as in Equation 1, and *d_ij_*(*h*) is the allelic dosage of founder *h* at marker locus *j* from Equation 2. The model includes eight coefficients *β_h_* representing the additive genetic effects of each founder haplotype. Thus the likelihood-ratio test has 7 degrees of freedom. We expect a significant gain in power and more precise estimation of the regression coefficients compared to the full model. However, we risk missing dominance interactions among founder haplotypes.

#### Additive SNP model:

To implement the additive SNP model we compute a probabilistic imputation of the genotype at every known SNP locus genome-wide (Baud *et al.* 2013). The expected allelic dosage is computed as

$$g_{ij}=\sum_{h=1}^{8}d_{ij}(h)G_{j}(s),$$ (4)

where *G_j_(s)* is the number of occurrences of the reference allele at marker locus *j* for an animal with allelic dosage of founder *h* at that locus. An advantage of mapping at the two-state SNP level is the potential to introduce dominance effects with one additional degree of freedom. We focus on the SNP-based additive model, which is widely used in human association mapping (Bush and Moore 2012). To gain computational efficiency we assign the diplotype state probability between each adjacent pair of genotyped markers to the average of the flanking diplotype state probabilities. Any sets of SNPs in the interval with identical strain distribution patterns among the eight founders are assigned identical values and we compute the regression once for each set of identical SNPs in an interval.

We express the regression equation at SNP locus *j* as

$$y_{i}=\sum_{k}x_{ik}a_{k}+g_{ij}\beta_{g}+\gamma_{i}+\varepsilon_{i},$$ (5)

where *y_i_*, *x_ik_*, *a_k_*, *γ_i_*, and *ε_i_* are the same as in Equation 1, *g_ij_* is generated by Equation 4, and *β_g_* is the additive effect of allelic substitution.

#### Kinship:

For each pair of DO animals, we computed the expected allele sharing of founder haplotypes based on the diplootype probabilities from Equation 2. Specifically, the kinship between sample *i* and *e* is

$$k_{ie}=\frac{1}{M}\sum_{j=1}^{M}\frac{\sum_{h=1}^{8}d_{ij}(h)\times d_{ej}(h)}{\sqrt{\sum_{h=1}^{8}d_{ij}{\left(h\right)}^{2}}\times \sqrt{\sum_{h=1}^{8}d_{ej}{\left(h\right)}^{2}}},$$ (6)

where *M* is the number of markers and *d_ij_(h)* is the allelic dosage of founder *h* for sample *i* at marker locus *j* (Equation 2). We have implemented genome scans using the leave-one-chromosome-out method (Cheng and Palmer 2013; Yang *et al.* 2014).

#### Implementation:

We extended the QTLRel software (v. 0.2-14) (Cheng *et al.* 2011) to fit any of the regression models described above. QTLRel estimates the variance scaling factor for the kinship correction for each phenotype. To provide an alternative method for fitting the mixed model regression we adapted the R package MatrixEQTL (Shabalin 2012). MatrixEQTL accepts any user-supplied matrix as the error covariance matrix and applies the same covariance structure to all phenotypes. This provides substantially faster computation for multiple phenotypes but the use of the same error covariance matrix for all phenotypes is an approximation.

#### Significance thresholds:

A Bonferroni correction can be used to obtain genome-wide thresholds. For MUGA the 0.05 Bonferroni corrected threshold is −log(p) > 5.2, and for MegaMUGA it is −log(p) > 6.0. The equivalent LOD thresholds are dependent on the sample size and choice of regression model. Bonferroni corrections are often too conservative and it is desirable to use a stringent but less conservative threshold based on permutation analysis (Churchill and Doerge 1994). However, the kinship structure of the DO population violates the usual exchangeability conditions (Churchill and Doerge 2008). Recent reports have suggested that by fitting the regression model without the kinship adjustment term to each permuted data set, one can obtain conservative thresholds for the mixed-model genome scan (Cheng and Palmer 2013). We validate this procedure using simulations to show that it provides expected control of the type I error rates in the DO. We used a significance threshold of p ≤ 0.05 to select significant mapping associations.

Support intervals for QTL localization were determined using a 95% Bayesian credible interval (Sen and Churchill 2001). The LOD curve is transformed by raising it to the power of 10 and a region covering 95% of the area under the transformed curve defines the support interval. The area under the curve is numerically approximated using trapezoids between the marker loci.

### Power simulations

We randomly sampled genomes from a set of marginal reconstructions of 1129 DO animals at outbreeding generation G8. We simulated phenotypes using sample sizes of 200, 400, 600, 800, and 1000 animals, with MAF of 1, 2, 3, and 4 and QTL effect sizes of 0.125, 0.25, 0.375, 0.5, 0.625, 0.75, and 1.0 in standardized units. The MAF refers to the number of founders that contribute the causative allele. Each combination of these parameters was simulated 1000 times. Phenotypes were simulated as the sum of a QTL effect plus a polygenic background component and an independent normal error component each with unit variance. Thus the polygenic and independent error variance components contribute equally to the total background variance. For each simulation, the QTL location was selected at random on the autosomal genome, minor allele status was assigned to the appropriate number of founder strains, and the genotype was determined by the diplotype of the marginal reconstruction. The QTL effect size was added to one homozygous class and subtracted from the other. We ran genome scans using the additive haplotype model and declared detection of the target QTL if the LOD score exceeded a genome-wide adjusted p ≤ 0.05 threshold. LOD thresholds were determined once for each set of simulations at a given sample size based on the maximal LOD score obtained on each of 1000 genome scans of phenotypes drawn independently from a normal (0,1) distribution. We tabulated power as the detection of significant QTL within 5 Mb of the simulated QTL location. Note that while the type I error rate is controlled genome-wide, power is calculated at a fixed locus and thus can fall below the nominal 0.05 level.

### Type I error simulations

We used the same set of DO genomes as in the power simulations above and mapped simulated phenotypes on the 19 autosomes. We selected a sample size *n* (200, 400, 600, 800, or 1000) and drew *n* DO genomes at random from our G8 animals. We simulated a null phenotype by sampling *n* values from a Gaussian distribution. We mapped the phenotype using an additive haplotype model with a kinship adjustment. We repeated this process 1000 times for each sample size and report the proportion of times in which a QTL was detected on any autosome at the p ≤ 0.05 significance thresholds. Significance thresholds for the X chromosome require special consideration (Broman *et al.* 2006) and have not yet been implemented in DOQTL.

### Phenotypes

DO mice (742 total, 410 females, 332 males) from outbreeding generations G4 and G5 were obtained from the Jackson Laboratory. Mice were housed in polycarbonate cages (50 in.^2^, 4–5 mice per cage) and fed NIH-31 6% fat mouse diet *ad libitum*. Acidified (pH 2.8–3.2) or chlorinated (10–15 ppm residual Cl) water was provided in water bottles. Mice were maintained on a 12 hr light/12 hr dark cycle. At ages ranging from 8 to 10 weeks, 50 ml of blood was collected via retro-orbital bleed and stored at 2–8° in K_2_EDTA. Neutrophil counts and other whole blood parameters were measured within 24 hr of collection on the Drew Scientific HemaVet 850 FS analyzer (Dallas, TX). Samples were processed in 15 batches of ∼50 animals each and some failed samples were removed; 5 mm of tail were collected for genotyping. These mice were a subset of the 3029 mice genotyped on the MUGA array. All procedures were reviewed and approved by the Jackson Laboratory’s Institutional Animal Care and Use committee (Protocol JW10001/JW202).

### Heritability

The narrow sense heritability of neutrophil counts in the 742 DO mice was estimated by fitting a mixed model with sex and phenotyping batch as fixed effects and the additive genetic matrix as a random effect (Clifford and Mccullagh 2006). The ratio of the additive genetic variance over the total phenotypic variance was used to calculate *h*^2^ (Visscher *et al.* 2008).

### Data and software availability

All data associated with this manuscript can be obtained at http://do.jax.org/. The DOQTL R package is available from Bioconductor (http://bioconductor.org/) (Gentleman *et al.* 2004). Genotyping data on the MUGA and MegaMUGA for the DO founders and F1s is available at ftp://ftp.jax.org/MUGA.

## Results

### Genome reconstructions

We computed diplotype state probabilities and marginal haplotype reconstructions for 4542 DO mice using allele-call and intensity-based methods for MUGA or MegaMUGA data. For each of 34 DO animals, samples from the same DNA isolation were run on the MUGA in separate batches on different dates. The mean Pearson correlation between the allelic dosages of replicate samples was 0.97 with a standard deviation of 0.05. The mean correlation between unrelated samples was 0.02 with a standard deviation of 0.05. Marginal genotype reconstructions were highly concordant—the vast majority of SNPs shared the same maximum probability diplotype state between repeated samples (minimum 63%, median 97%, and maximum 99%). Discordant diplotype states occurred most often in regions where one or more of the founder strains are identical by state and as a result, two or more diplotype states have very similar probabilities.

**Figure 2:**
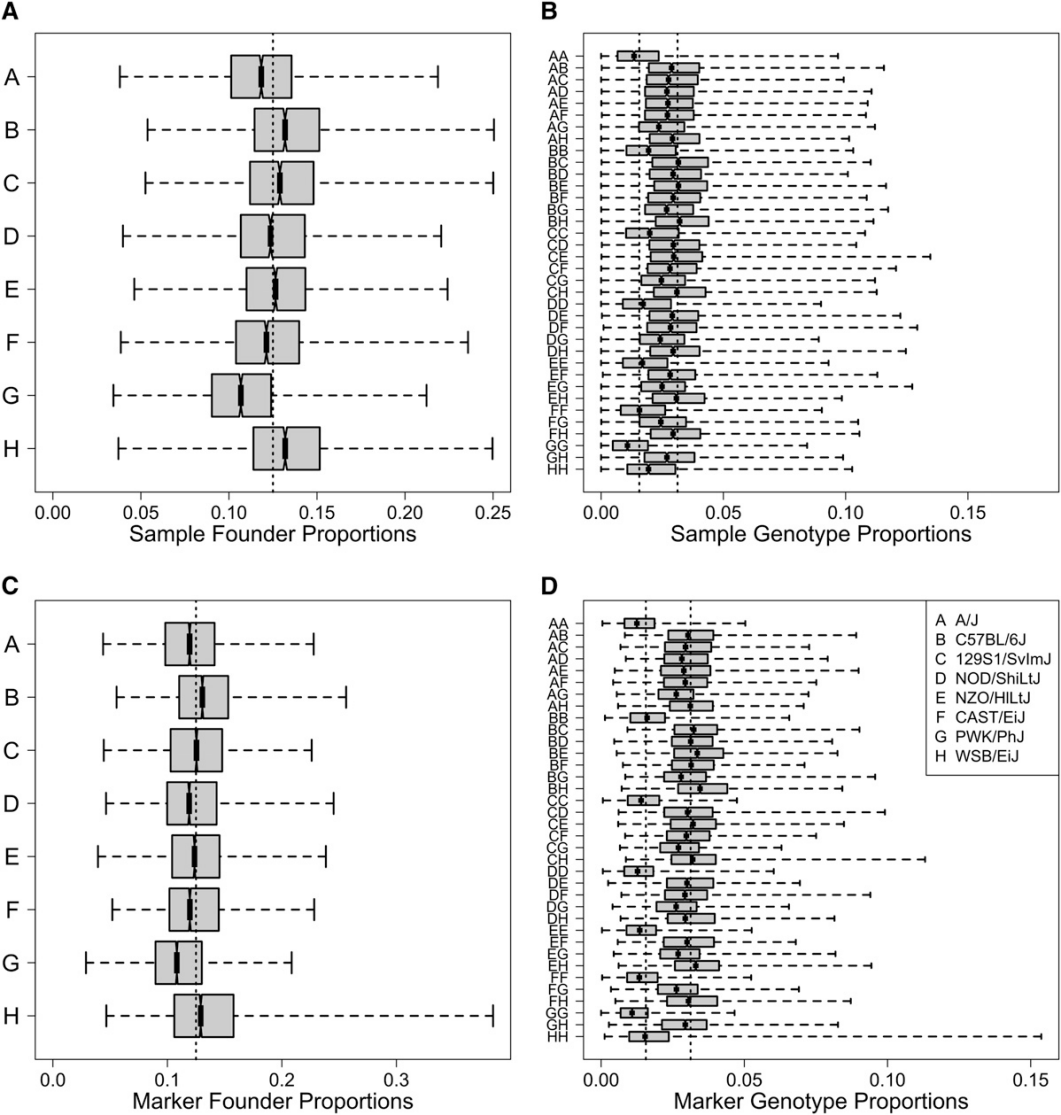
(A) Founder allele proportions across samples and markers. Horizontal axis shows the proportion of alleles from each founder across all samples. Boxes show median and interquartile range. Dotted line is 1/8. (B) Diplotype state proportions across all samples. Diplotypes on vertical axis are given in two-letter codes shown in legend. Dotted lines at 1/32 and 1/64. (C) Founder allele proportions across markers. Dashed line is a 1/8. (D) Diplotype state across markers.

We used marginal haplotype reconstructions to compute the proportion of each founder summed across markers for each DO animal (Figures 2, A and B). The median founder contribution ranged from 10.6% (PWK/PhJ) to 13.2% (C57BL/6J)—consistent with an expectation of 12.5%. Individual DO animals displayed a wide range of founder contributions with a minimum of 3.4% and a maximum of 25.1%. The proportions of the 36 diplotypes are also consistent with expectations—each heterozygous diplotype should occur at a frequency of 1/32 and each homozygous diplotype should occur at a frequency of 1/64. The only notable deviation is an underrepresentation of homozygous PWK/PhJ genotype, which occurs at a median frequency of 0.011%, which is consistent with the lower overall contribution of PWK/PhJ haplotypes. We also computed the contribution of each founder strain at individual SNPs summed across all of the DO mice (Figures 2, C and D). A notable deviation from expected frequencies occurs on chromosome 2 where a previously reported excess of WSB/EiJ founder allele is observed (Svenson *et al.* 2012).

**Figure 3:**
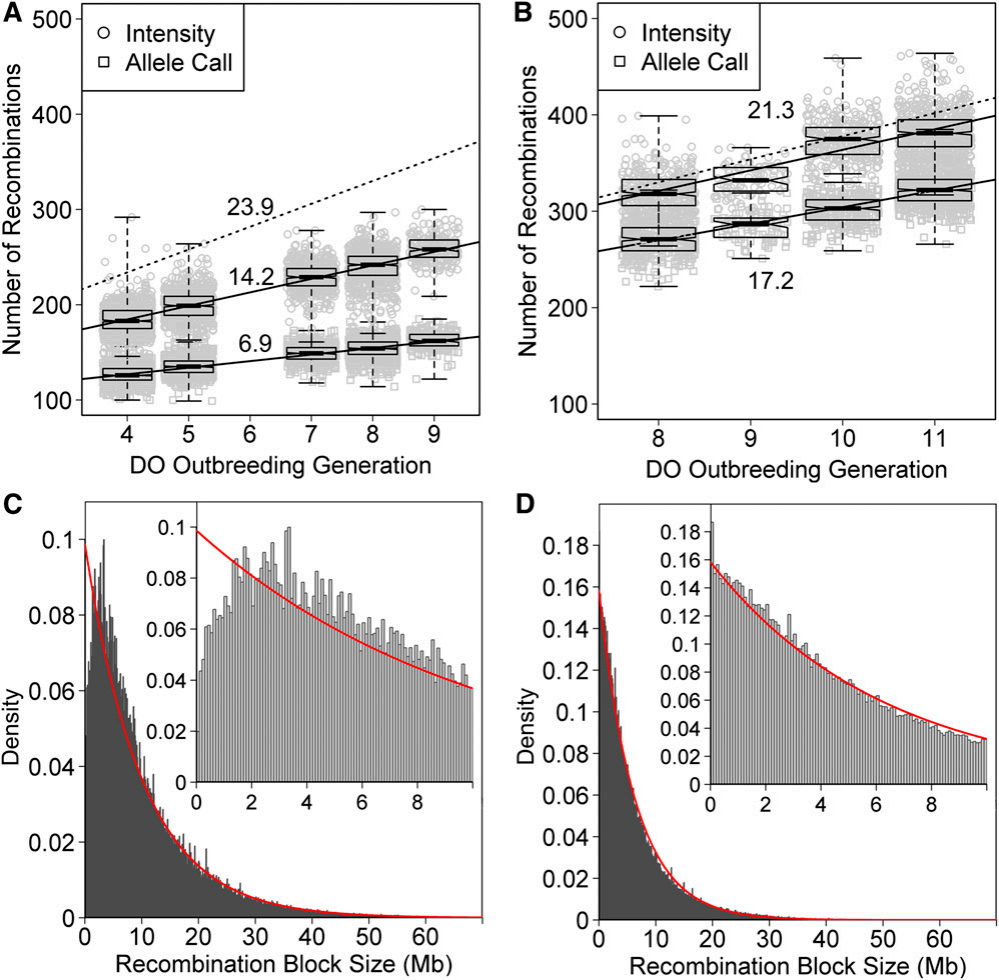
(A) Estimated number of autosomal recombinations per sample run on the MUGA plotted by generation using the marker allele calls (□) or the genotyping array intensities (○). Solid lines are the least-squares fit for each method. Dashed line is the expected theoretical number of recombinations. Numbers are the slopes of each line. Boxes show median plus interquartile range. (B) The same plot as in A, but with samples run on the MegaMUGA. Top and bottom numbers are the slope of the intensity- and allele-call-based reconstructions, respectively. (C) Histogram of recombination block size using the MUGA (C) and MegaMUGA (D). The inset shows the region from 0 to 10 Mb. Red curves are the maximum-likelihood fit of the data to an exponential distribution.

The average number of autosomal recombination events in DO mice is expected to increase linearly at a rate of 23.9 events per generation (Broman 2012). We compared this rate between the intensity-based and genotype-call methods of haplotype reconstruction on each of the MUGA and MegaMUGA genotyping platforms. For MUGA samples spanning generations G4 through G9 we observed a rate of increase of 14.2 events per generation using the intensity-based method compared to 6.9 events per generation for the allele-call method (Figure 3A). For MegaMUGA samples spanning generations G8 through G11 we observed a rate of increase of 21.3 events per generation using the intensity-based and 17.2 events per generation for the allele-call method (Figure 3B). The intensity-based MegaMUGA data give estimates that are close to the theoretical rate, suggesting that recombination events are accumulating as expected and that the other methods are not capturing all of the events. For both platforms, the intensity-based method captures more recombination events compared to the allele call but the difference is greater for the MUGA platform. The average marker spacing of the MUGA (325 kb) is large relative to the expected minimum spacing of ∼40 kb at generation G8 (Pyke 1965), whereas MegaMUGA, with an average marker spacing of 33.2 kb, should capture most of the recombination events in current DO generations. We note that the size distribution of haplotype segments should approximate an exponential distribution. However, histograms of interval sizes on the MUGA array (Figure 3C) show a drop in the numbers of small segments relative to their expected distribution at sizes below the mean marker spacing, indicating that small haplotype blocks may be lost in the reconstructions. The closer marker spacing on the MegaMUGA helps to increase the rate of detecting small recombination blocks (Figure 3D).

### Simulations

Null model simulations established that type I error rates are correctly controlled for permutation-based thresholds (Cheng and Palmer 2013). We simulated 1000 phenotypes by sampling from a multivariate normal distribution with a covariance structure equal to the kinship matrix between samples. We permuted the phenotype values 1000 times and retained the maximum LOD score across the 19 autosomes to obtain the empirical distribution of maximum LOD scores. Quantile plots of the type I error indicate that all three regression models described in *Materials and Methods* control the type I error at or below the desired threshold (Supporting Information, Figure S1). In particular, the type I error at *α* = 0.05 is properly controlled. The computational time required to fit a linear mixed-model genome scan repeated for each permuted data sample can be substantial. Thus our ability to obtain valid thresholds with a simple linear model fit has practical importance.

**Figure 4:**
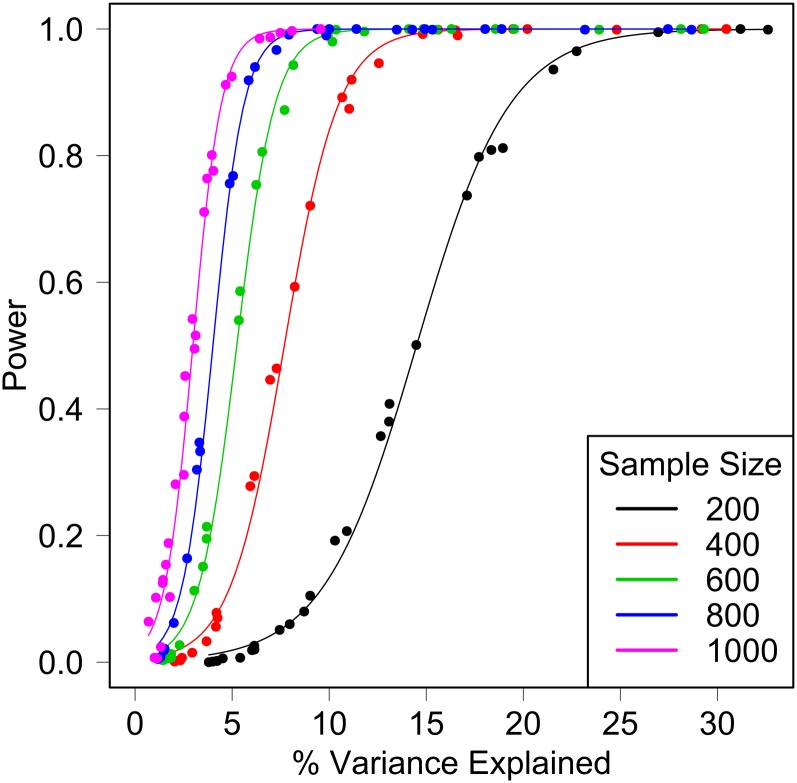
Power simulations demonstrate the relationship between power, sample size, and percentage variance explained. The percentage variance explained by the simulated QTL is plotted *vs.* the power to detect the simulated QTL for five different sample sizes. Points are the mean value from 1000 simulations and curves are logistic regression models fit to the data.

Power simulations provide guidelines for selecting an appropriate sample size for experiments. The power to detect a QTL depends on its effect size, the residual and genetic background variances, and the frequency of the causative allele. These factors are difficult to determine in advance but simulations can help to establish expectations for a study of a given size. We simulated QTL over a wide range of effect sizes with minor allele frequencies that range from 1/8 to 1/2. We fixed the ratio of genetic to residual variation to be one—equal contributions of each—and scaled effect sizes relative to the residual standard deviation. We show results for the additive haplotype model (Equation 3) and find, as expected, that increasing MAF, effect size, or the sample size increases power (Table S1). The percentaage variance explained provides an effective summary of the combined effects of these factors on power (Figure 4). With a sample size of 200 mice, we can expect to detect QTL that explain >20% of the phenotypic variance with 90% power. With a sample of 1000 mice, we can expect to detect a QTL that explain 5% of the variance with 90% power.

We examined power of the full model and of the additive SNP model using simulations with a sample size of 600, a MAF of 2, and an effect size of 0.5 standard deviations from the mean. A QTL with these characteristics will account for 9% of the phenotypic variance. We found the power of the full model to be 0.003 and the type I error at *α* = 0.05 to be 0.046. For the additive haplotype model, power was 0.94 and the type I error was 0.051. For the additive SNP model, power was 0.91 and the type I error was 0.04. These limited simulations indicate that the size of the test is controlled at the nominal genome-wide *P* ≤ 0.05 level. There is a dramatic loss of power using the full model, whereas, for data simulated under the additive SNP model, the additive haplotype and additive SNP models have similar power. This similarity in power for the additive SNP model represents a best-case scenario because we simulated a biallelic, additive QTL. When allelic heterogeneity is present under the QTL, the power of the additive SNP model may be lower.

Larger sample sizes and larger effect sizes also provide narrower QTL intervals (Table S2). Support intervals can range (on average) from 5 Mb for effects that explain 5% of the variance down to <1 Mb for effects that explain 30% of the variance in mapping population of 1000 mice.

### Example: QTL mapping of neutrophil counts

Here we illustrate the unique features of QTL mapping in the DO with an example: mapping neutrophil counts in whole blood using 742 DO mice (410 female, 332 male). Males [median, 1004; interquartile range,IQR, (698, 1288)] had higher median neutrophil counts than females [median, 729; IQR, (546, 1064))]. The trait has an estimated heritability of 0.132.

**Figure 5:**
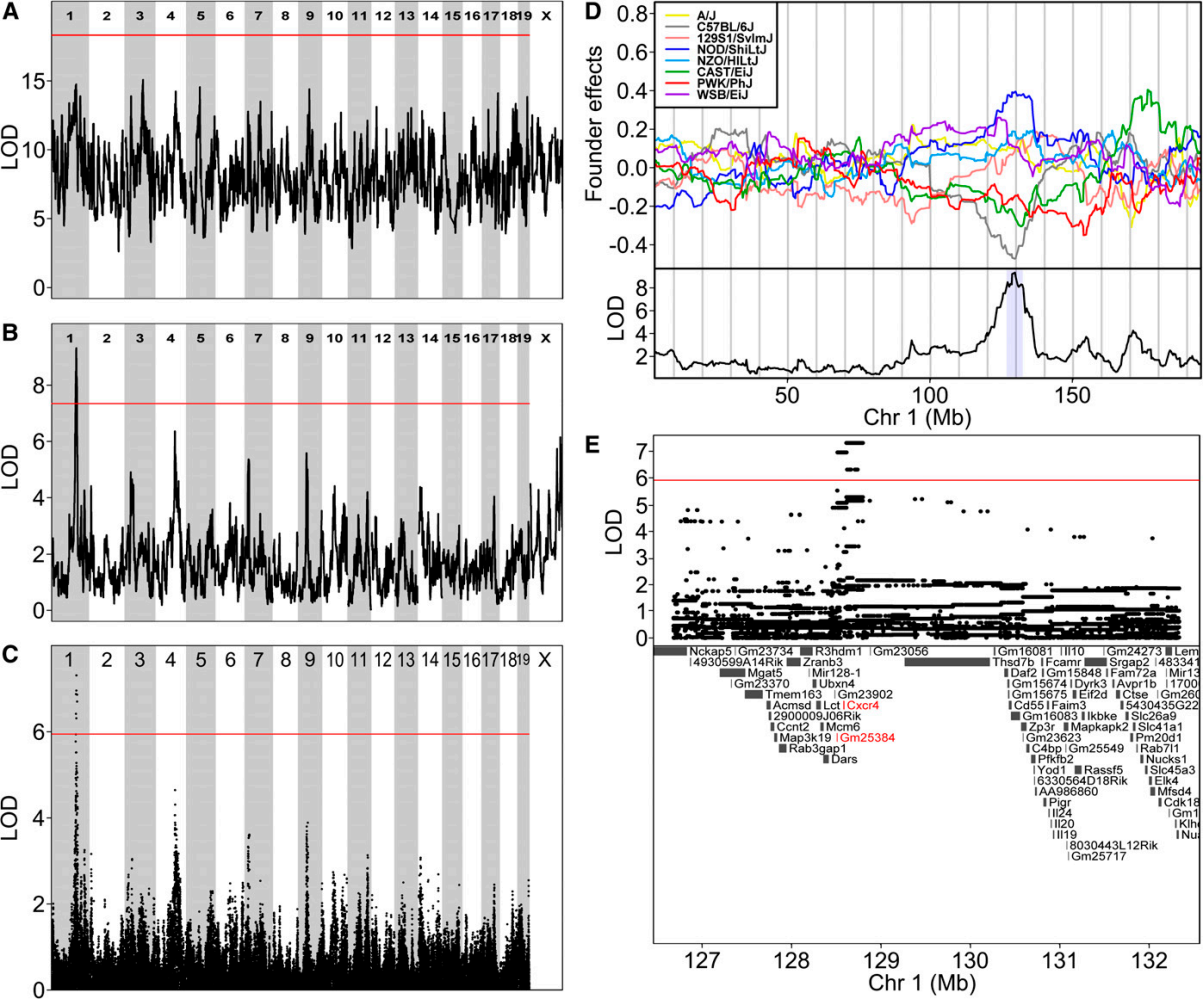
Mapping of constitutive neutrophil counts. (A) Genome scan of constitutive neutrophil counts using the full model does not show any significant peaks. The horizontal axis shows the mouse genome. The vertical axis plots the LOD score at each locus. Red line is p ≤ 0.05 significance threshold. (B) Linkage mapping of neutrophil counts using the additive haplotype model reveals a large peak on chromosome 1. (C) Association mapping of neutrophil counts using the additive SNP model produces a plot similar to linkage mapping. Vertical axis shows LOD score. Red line is p ≤ 0.05 significance threshold. (D) Founder coefficients from the linkage model on chromosome 1 show the effects of each founder allele. The founder coefficients are centered around zero. Bottom shows the LOD score with support interval shaded light blue. (E) Zoomed view of QTL support interval highlights two genes (GRC Build 38 coordinates). Top shows the LOD score for the additive SNP model with the red line denoting the *P* ≤ 0.05 significance threshold.

We regressed log neutrophil counts on founder allele dosages at each marker using a kinship correction with sex and log white blood cell counts as covariates. We established genome-wide significance thresholds as described in *Materials and Methods*. We fit the full model (Equation 1) by regressing the log of neutrophil counts on the 36 diplotype probabilities at each marker and found that no LOD peaks exceeded the significance threshold (adjusted p ≤ 0.05) (Figure 5A). We fit the additive haplotype model (Equation 3) and found that one locus on chromosome 1 (LOD, 9.3 at 129.58 Mb GRC Build 38) achieved genome-wide significance and spanned a 5.7-Mb support interval (126.7–132.4 Mb, Figure 5B). We fit an additive SNP model (Equation 5) by regressing the log of neutrophil counts on the imputed DO genotypes and found one locus with LOD above the p ≤ 0.05 threshold on chromosome 1 (Figure 5C). In the additive SNP model, the peak on chromosome 1 is still the largest peak. The additive haplotype model produces estimates of the eight founder allele effects (Figure 5D). The founder allele effects separate into two groups and the peak may be due to a single diallelic polymorphism, which would be consistent with the additive SNP model. In this case, DO mice containing the C57BL/6J, CAST/EiJ, or PWK/PhJ alleles at the QTL on chromosome 1 have lower neutrophil counts than mice containing the other five founder alleles.

We examined the mapping results from the additive SNP model in the QTL support interval on chromosome 1 and found that two types of SNPs had LOD greater than the p ≤ 0.05 significance threshold (Figure 5E). Together these SNPs span an interval from 128.519324 to 128.801978 Mb and overlap two annotated features: *Gm25384* and chemokine receptor 4 (*Cxcr4*). *Gm25384* is a noncoding RNA with no polymorphisms in the DO. *Cxcr4* has three SNPs in the coding region of its transcript, two synonymous SNPs (no rs#, rs8256193), and one nonsynonymous SNP (rs8256191, 128.589277 Mb, C → T). The minor allele at each of these three SNPs distinguishes strains C57BL/6J, CAST/EiJ, and PWK/PhJ from the remaining founders, consistent with the estimated effects from the additive haplotype model in Figure 5D. *Cxcr4* has two alternative transcripts and the nonsynonymous SNP is in an exon shared by both isoforms. One of the synonymous variants (no rs#) at 128.589098 Mb occurs in one of the isoforms. The other synonymous variant (rs8256193) occurs in both isoforms. In addition, the founder strains C57BL/6J and PWK/PhJ share a segment of common ancestry (130.414213–130.698555 Mb) from the *Mus mus musculus* progenitor (http://csbio.unc.edu/ccv/) (Yang *et al.* 2011). *Cxcr4* has been shown to play a role in regulation of neutrophil release from the bone marrow (Eash *et al.* 2009), making it a strong functional candidate. On the basis of this evidence we suggest that *Cxcr4*, specifically the valine to isoleucine substitution, is a causal factor contributing to variation in neutrophil levels.

## Discussion

Haplotype reconstruction in progeny from multiparent crosses requires specialized methods and software. These methods have evolved significantly over the past decade and we expect to see a continued increase in the overlap between heterozygous model systems and human genetic mapping. HAPPY, one of the first software packages developed for haplotype reconstruction in heterogeneous stocks, uses genotype calls and a dynamic programming algorithm for haplotype reconstruction (Mott *et al.* 2000). Our HMM implementation is more complex than dynamic programming but it provides probabilistic estimates of haplotypes (and diplotypes) that we can use to account for genotype uncertainty in genome scans. The current implementation is based on the marginal diplotype state probabilities. Implementation of a globally optimal reconstruction (Van der Bliek *et al.* 1988; Viterbi 1967) is planned for a future release of DOQTL.

We have implemented an EM algorithm to estimate HMM parameters directly from genotype or intensity data. The direct use of probe intensities from genotyping arrays can improve genome reconstructions (Fu 2012). Probes that are affected by off-target variants and might otherwise be removed by genotyping quality filtering can provide additional information about founder haplotypes (Didion *et al.* 2012). Our emission model for intensities is heavily parameterized as a bivariate normal mixture model over 36 diplotype states at each marker locus. It is likely that performance could be improved by adding constraints to this model, for example, by leveraging haplotype identity among founder strains to reduce the number of states required at each SNP or by modeling heterozygous genotypes in terms of the homozygous state intensities. Our state transition model is not estimated; it is derived from theoretical expectations as a function of the outcrossing generation in the DO. In more complex breeding designs, this may not be feasible and an empirical method to estimate the transition probabilities would be desirable. One approach might be to use a continuous “time” Markov model with transition probabilities p ≤ *e^Qt^*, where *Q* is a common 36 × 36 rate matrix and *t* is in an interval-specific measure of recombination. As with the emission model, there is a bias *vs.* variance tradeoff to consider in the specification of these highly parameterized models. Models that capture the main features of the data with the fewest parameters are best.

Our estimates of the number of recombination events per mouse are close to the theoretical expectation when we used the higher density MegaMUGA array and intensity based reconstruction. The MegaMUGA includes >77,000 informative markers with average spacing <33 kb. Still it is clear that smaller haplotype blocks are lost in the reconstructions. The economics of using even higher density arrays platform *vs.* low coverage whole-genome sequencing is starting to tip. New analytical methods will be required for genotypes derived from sequencing data. The DO is currently at outbreeding generation G17 and the accumulation of recombination events appears to be linear as expected through generation G11. Hotspots of recombination of 1–2 kb in size can have recombination rates as high as 2–3 cM (Billings *et al.* 2010; Parvanov *et al.* 2010). Recurrent recombination events at hotspots could impose a limit on the ultimate mapping resolution of the DO. In addition, cold regions of >500 kb with strongly suppressed recombination are present in the DO (data not shown) and are largely concordant with recent reports of cold regions in the founder strains (Liu *et al.* 2014).

Availability of whole-genome sequences of the founder strains adds substantially to the power and utility of multiparent populations (Baud *et al.* 2013). With dense genotypes and haplotype reconstructions we can effectively impute the entire genome of each individual and track all of the possible causal variants associated with any phenotype. SNPs can be imputed using either marginal or probabilistic genotypes. The same is true for small indels but larger structural and copy-number variations present some unsolved challenges. Structural variants could potentially be changing rapidly in multiparent populations, providing a unique opportunity to study their short-term evolution.

We considered three different regression models with which to perform mapping in the DO: a full model, an additive haplotype model, and an additive SNP model. When mapping using the full model, we observed loss of power that we attribute to the additional degrees of freedom. The additive haplotype and SNP models can produce similar results. A number of open questions about optimal strategies to model QTL effects on phenotypes in multiparent crosses remain. Our work looks at the extremes of a spectrum of possible models. The challenge is to develop models that are flexible and yet retain sufficient power to detect QTL. Random effects or hierarchical Bayes models look promising in this regard (Zhaojun *et al.* 2014).

One advantage of linkage mapping in multiparent populations such as the DO is that we obtain estimates of the founder allele effects at each locus. The pattern of allele effects and sequencing data from the eight founders were critical in narrowing the neutrophil QTL to a single gene. For some QTL the allele effects split into two clearly delineated groups, *e.g.*, C57BL/6J, CAST/EiJ, and PWK/PhJ *vs.* the remaining five founders for the neutrophil QTL on chromosome 1. This is expected if the QTL is caused by a single variant. We have also observed cases where the allele effects split into multiple classes or where the classification of effects into discrete clusters is not clear. These patterns likely result from multiple closely linked causal variants. Allele-effect patterns can help to establish whether comapping of multiple phenotypes is due to the same variant(s) or linked but independent variants (King *et al.* 2012).

DO mice, after multiple generations of outcrossing, will have complex kinship relationships, including siblings, cousins, and extended multigenerational relationships of unknown degree. These relationships must be accounted for in the mapping analysis or increases in false positives may be observed (Cheng *et al.* 2013). Methods for kinship correction remain an active area of research (Zhou and Stephens 2012; Yang *et al.* 2014).

We recommend using a larger number of animals for a DO mapping study than is typical for mapping studies in the mouse. In our experience loci of major effect have been precisely mapped with <200 animals. It would be prudent to plan on large studies when using the DO to investigate complex traits. The DO provides substantial advantages, as illustrated in the above QTL mapping example.

The DOQTL software implements the methods described in this article from reconstruction of haplotypes through QTL mapping and graphical summaries and reports. With a few modifications, DOQTL can be used to analyze other multiparent populations (Kover *et al.* 2009) and heterogeneous stock populations (Baud *et al.* 2013). We encourage investigators who wish to apply our software to analyze their populations to contact us. Tutorials and additional supporting materials are available at http://do.jax.org. This site will serve as a repository for DO related data and we encourage groups using the DO to contribute their published data.

## Supplementary Material

Supporting Information
