# Supplementary material for: Quantitative Trait Locus Mapping Methods for Diversity Outbred Mice
Source: G3 (Bethesda). 2014 Sep 1;4(9):1623–33. doi: 10.1534/g3.114.013748 (PMC4169154; doi:10.1534/g3.114.013748)
Supplement: Supporting Information [file supp_4_9_1623__index.html]

Supporting Information 

# Quantitative Trait Locus Mapping Methods for Diversity Outbred Mice

## Supporting Information for Gatti *et al.*, 2014

**Files in this Data Supplement:**

- Supporting Information - Figure S1 and Tables S1 and S2 (PDF, 460 KB)
- Figure S1 - Quantile-quantile plots of the type I error for the full model (PDF, 278 KB)
- Table S1 - Power Simulations (PDF, 135 KB)
- Table S2 - Mean QTL width across simulations using DO mice from outbreeding generation 8 (PDF, 124 KB)
