# Supplementary material for: Quantitative Trait Locus Mapping Methods for Diversity Outbred Mice
Source: G3 (Bethesda). 2014 Sep 1;4(9):1623–33. doi: 10.1534/g3.114.013748 (PMC4169154; doi:10.1534/g3.114.013748)
Supplement: Supporting Information [file supp_4.9.1623_161844SI.pdf]

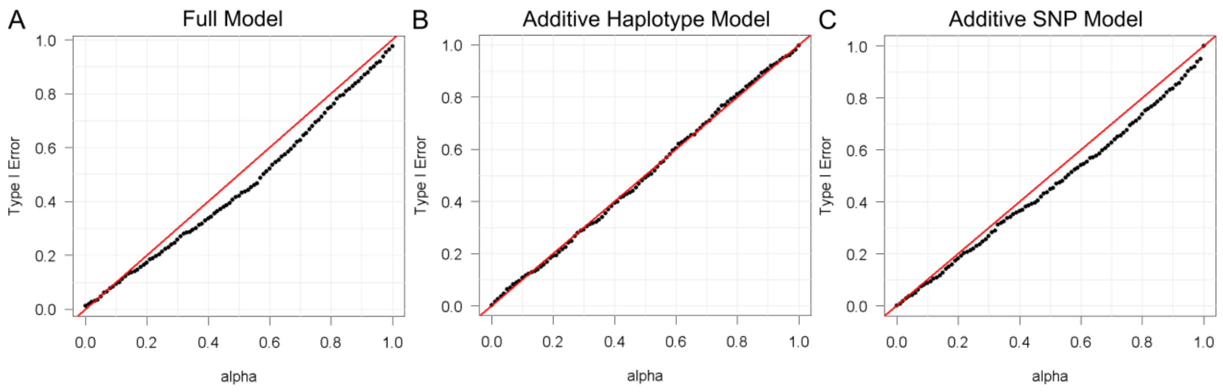

Figure S1 Quantile-quantile plots of the type I error for the full model (A), the additive haplotype model (B) and the additive SNP model (C). The type I error is well controlled at low values of alpha and is slightly conservative at higher values.

Table S1 Power Simulations. Each cell reports the proportion of simulations in which a simulated QTL was detected at a permutation derived $p_{GW} \leq 0.05$. Effect size is the number of standard deviations from the mean.

	MAF 1/8					MAF 2/8				
**Effect Size**	**200**	**400**	**600**	**800**	**1000**	**200**	**400**	**600**	**800**	**1000**
**0.125**	0	0.001	0.003	0.005	0.007	0.001	0.003	0.006	0.008	0.006
**0.25**	0.006	0.015	0.027	0.062	0.103	0.007	0.033	0.113	0.164	0.296
**0.375**	0.02	0.078	0.214	0.347	0.516	0.051	0.278	0.54	0.756	0.899
**0.5**	0.06	0.294	0.586	0.768	0.903	0.207	0.721	0.943	0.991	0.999
**0.625**	0.192	0.593	0.872	0.967	0.987	0.501	0.946	0.996	1	0.999
**0.75**	0.408	0.874	0.98	0.99	0.999	0.809	0.998	0.999	1	0.999
**1**	0.812	0.99	1	0.999	0.999	0.995	0.999	0.999	0.999	1

	MAF 3/8					MAF 4/8				
**Effect Size**	**200**	**400**	**600**	**800**	**1000**	**200**	**400**	**600**	**800**	**1000**
**0.125**	0.002	0.007	0.007	0.018	0.019	0.002	0.003	0.013	0.021	0.024
**0.25**	0.019	0.056	0.151	0.304	0.476	0.026	0.07	0.195	0.333	0.495
**0.375**	0.08	0.446	0.754	0.919	0.928	0.105	0.464	0.806	0.94	0.989
**0.5**	0.357	0.892	0.989	1	0.987	0.38	0.92	0.999	1	1
**0.625**	0.737	0.992	1	0.999	0.998	0.798	0.999	1	0.999	1
**0.75**	0.936	1	1	1	0.999	0.965	1	1	1	0.999
**1**	1	1	1	1	1	0.999	1	1	0.999	0.999

Table S2 Mean QTL width across simulations using DO mice from outbreeding generation 8.

Each cell represents the mean QTL width when a QTL was detected. Blank cells indicate that QTL were detected less than 10% of the time.

	MAF 1/8					MAF 2/8				
**Effect Size**	**200**	**400**	**600**	**800**	**1000**	**200**	**400**	**600**	**800**	**1000**
**0.125**	--	--	--	--	--	--	--	--	--	--
**0.25**	--	--	--	-	9.60	--	--	5.66	5.54	6.00
**0.375**	--	--	6.53	8.01	6.10	--	5.81	5.69	5.09	3.82
**0.5**	--	5.81	5.42	4.48	3.88	6.87	4.85	3.56	2.60	2.04
**0.625**	6.26	4.85	3.74	2.98	2.45	5.28	3.42	2.13	1.75	1.56
**0.75**	6.66	4.02	2.69	2.24	1.86	4.70	2.25	1.64	1.39	1.28
**1**	4.05	2.39	1.77	1.48	1.30	2.56	1.56	1.17	1.05	1.10

	MAF 3/8					MAF 4/8				
**Effect Size**	**200**	**400**	**600**	**800**	**1000**	**200**	**400**	**600**	**800**	**1000**
**0.125**	--	--	--	--	--	--	--	--	--	--
**0.25**	--	--	7.73	7.48	6.68	--	--	6.78	5.46	6.71
**0.375**	--	6.36	5.29	3.71	2.27	7.05	5.99	5.06	3.40	2.79
**0.5**	8.15	3.96	2.75	2.00	1.85	5.74	4.01	2.50	1.95	1.60
**0.625**	5.20	2.53	1.78	1.44	1.57	5.25	2.42	1.65	1.39	1.24
**0.75**	3.66	1.74	1.37	1.17	1.02	3.21	1.75	1.29	1.14	1.09
**1**	2.13	1.29	1.04	0.98	0.88	1.95	1.20	1.02	0.89	0.86
