# Supplementary material for: Quantitative Trait Locus Mapping Methods for Diversity Outbred Mice
Source: G3 (Bethesda). 2014 Sep 1;4(9):1623–33. doi: 10.1534/g3.114.013748 (PMC4169154; doi:10.1534/g3.114.013748)
Supplement: Supporting Information [file supp_4.9.1623_FigureS1.pdf]

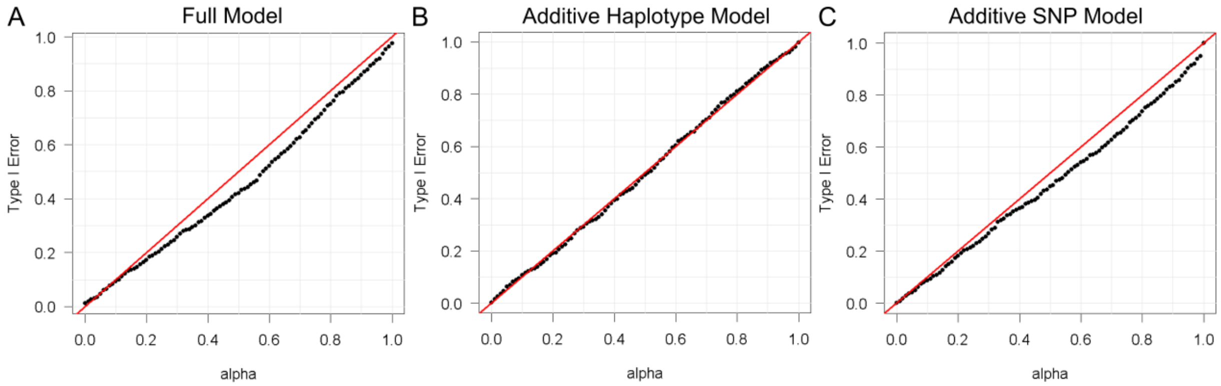

Figure S1 Quantile-quantile plots of the type I error for the full model (A), the additive haplotype model (B) and the additive SNP model (C). The type I error is well controlled at low values of alpha and is slightly conservative at higher values.
