# Supplementary material for: Quantitative Trait Locus Mapping Methods for Diversity Outbred Mice
Source: G3 (Bethesda). 2014 Sep 1;4(9):1623–33. doi: 10.1534/g3.114.013748 (PMC4169154; doi:10.1534/g3.114.013748)
Supplement: Supporting Information [file supp_4.9.1623_TableS1.pdf]

Table S1 Power Simulations. Each cell reports the proportion of simulations in which a simulated QTL was detected at a permutation derived $p_{GW} \leq 0.05$. Effect size is the number of standard deviations from the mean.

	MAF 1/8					MAF 2/8				
**Effect Size**	**200**	**400**	**600**	**800**	**1000**	**200**	**400**	**600**	**800**	**1000**
**0.125**	0	0.001	0.003	0.005	0.007	0.001	0.003	0.006	0.008	0.006
**0.25**	0.006	0.015	0.027	0.062	0.103	0.007	0.033	0.113	0.164	0.296
**0.375**	0.02	0.078	0.214	0.347	0.516	0.051	0.278	0.54	0.756	0.899
**0.5**	0.06	0.294	0.586	0.768	0.903	0.207	0.721	0.943	0.991	0.999
**0.625**	0.192	0.593	0.872	0.967	0.987	0.501	0.946	0.996	1	0.999
**0.75**	0.408	0.874	0.98	0.99	0.999	0.809	0.998	0.999	1	0.999
**1**	0.812	0.99	1	0.999	0.999	0.995	0.999	0.999	0.999	1

	MAF 3/8					MAF 4/8				
**Effect Size**	**200**	**400**	**600**	**800**	**1000**	**200**	**400**	**600**	**800**	**1000**
**0.125**	0.002	0.007	0.007	0.018	0.019	0.002	0.003	0.013	0.021	0.024
**0.25**	0.019	0.056	0.151	0.304	0.476	0.026	0.07	0.195	0.333	0.495
**0.375**	0.08	0.446	0.754	0.919	0.928	0.105	0.464	0.806	0.94	0.989
**0.5**	0.357	0.892	0.989	1	0.987	0.38	0.92	0.999	1	1
**0.625**	0.737	0.992	1	0.999	0.998	0.798	0.999	1	0.999	1
**0.75**	0.936	1	1	1	0.999	0.965	1	1	1	0.999
**1**	1	1	1	1	1	0.999	1	1	0.999	0.999
