# Supplementary material for: Quantitative Trait Locus Mapping Methods for Diversity Outbred Mice
Source: G3 (Bethesda). 2014 Sep 1;4(9):1623–33. doi: 10.1534/g3.114.013748 (PMC4169154; doi:10.1534/g3.114.013748)
Supplement: Supporting Information [file supp_4.9.1623_TableS2.pdf]

**Table S2 Mean QTL width across simulations using DO mice from outbreeding generation 8.**

Each cell represents the mean QTL width when a QTL was detected. Blank cells indicate that QTL were detected less than 10% of the time.

| | MAF 1/8 | | | | | MAF 2/8 | | | | |
|---|---|---|---|---|---|---|---|---|---|---|
| **Effect Size** | **200** | **400** | **600** | **800** | **1000** | **200** | **400** | **600** | **800** | **1000** |
| **0.125** | -- | -- | -- | -- | -- | -- | -- | -- | -- | -- |
| **0.25** | -- | -- | -- | - | 9.60 | -- | -- | 5.66 | 5.54 | 6.00 |
| **0.375** | -- | -- | 6.53 | 8.01 | 6.10 | -- | 5.81 | 5.69 | 5.09 | 3.82 |
| **0.5** | -- | 5.81 | 5.42 | 4.48 | 3.88 | 6.87 | 4.85 | 3.56 | 2.60 | 2.04 |
| **0.625** | 6.26 | 4.85 | 3.74 | 2.98 | 2.45 | 5.28 | 3.42 | 2.13 | 1.75 | 1.56 |
| **0.75** | 6.66 | 4.02 | 2.69 | 2.24 | 1.86 | 4.70 | 2.25 | 1.64 | 1.39 | 1.28 |
| **1** | 4.05 | 2.39 | 1.77 | 1.48 | 1.30 | 2.56 | 1.56 | 1.17 | 1.05 | 1.10 |

| | MAF 3/8 | | | | | MAF 4/8 | | | | |
|---|---|---|---|---|---|---|---|---|---|---|
| **Effect Size** | **200** | **400** | **600** | **800** | **1000** | **200** | **400** | **600** | **800** | **1000** |
| **0.125** | -- | -- | -- | -- | -- | -- | -- | -- | -- | -- |
| **0.25** | -- | -- | 7.73 | 7.48 | 6.68 | -- | -- | 6.78 | 5.46 | 6.71 |
| **0.375** | -- | 6.36 | 5.29 | 3.71 | 2.27 | 7.05 | 5.99 | 5.06 | 3.40 | 2.79 |
| **0.5** | 8.15 | 3.96 | 2.75 | 2.00 | 1.85 | 5.74 | 4.01 | 2.50 | 1.95 | 1.60 |
| **0.625** | 5.20 | 2.53 | 1.78 | 1.44 | 1.57 | 5.25 | 2.42 | 1.65 | 1.39 | 1.24 |
| **0.75** | 3.66 | 1.74 | 1.37 | 1.17 | 1.02 | 3.21 | 1.75 | 1.29 | 1.14 | 1.09 |
| **1** | 2.13 | 1.29 | 1.04 | 0.98 | 0.88 | 1.95 | 1.20 | 1.02 | 0.89 | 0.86 |
